# Editorial: Community series in: cardiovascular risks in cardiovascular-kidney-metabolic syndrome: mechanisms and therapies, volume II

**DOI:** 10.3389/fendo.2026.1966328

**Published:** 2026-08-27

**Authors:** Ruoyu Zhou, Bo Zhang, Weihao Wang, Jiyuan Piao, Wanlu Ma

**Affiliations:** 1 Department of Endocrinology, China-Japan Friendship Hospital, Beijing, China; 2 Department of Endocrinology, Beijing Hospital, Beijing, China; 3 Faculty of Medicine &Dentistry-Medicine Department, University of Alberta, Edmonton, AB, Canada

**Keywords:** CKM syndrome, end-organ injury, mechanism, metabolic dysfunction, therapy

The emergence of cardiovascular–kidney–metabolic (CKM) syndrome represents more than the creation of a new clinical classification system, it reflects a fundamental change in how we understand chronic cardiometabolic disease (1, 2). Obesity, type 2 diabetes, cardiovascular disease, and chronic kidney disease — long treated as related but largely separate problems — are now recognized as facets of a single, evolving biological network driven by insulin resistance, oxidative stress, lipotoxicity, chronic inflammation, and renin–angiotensin–aldosterone system (RAAS) dysregulation (1, 3). The journey most often begins with dysfunctional visceral adipose tissue, whose proinflammatory and prooxidant secretions injure the vasculature, myocardium, and kidneys while impairing insulin sensitivity (4, 5). Despite longstanding recognition of the cardiovascular burden carried by diabetes and the metabolic syndrome, the specific mechanisms through which CKM syndrome amplifies cardiovascular risk remain incompletely understood. This Research Topic — Community Series in: Cardiovascular Risks in Cardiovascular-Kidney-Metabolic Syndrome: Mechanisms and Therapies - Volume II— aims to elucidate these mechanisms, from metabolic drivers through vascular and renal injury to inflammation and end-organ damage, and to identify potential therapeutic targets along this causal chain. The 13 original articles, reviews, and clinical studies assembled here examine that chain link by link: from the upstream metabolic trigger, through vascular–renal bridging and immune–inflammatory amplification, to downstream interventions and clinical outcomes.

## Upstream: metabolic dysfunction as the point of ignition

Metabolic dysregulation is the starting point of the CKM trajectory, and three studies in this section illuminate how it drives renal injury long before structural damage becomes apparent. A cross-sectional comparison of Chinese and U.S. populations found that an insulin resistance/dyslipidemia score, rather than visceral adiposity or inflammation alone, was the strongest correlate of established CKM status. Most of the visceral adiposity effect was statistically attributable to this metabolic axis. Systemic inflammation, by contrast, became prominent only in advanced disease (Zhang et al.). Longitudinal evidence then confirmed the direction of this relationship: a higher TyG index, a practical surrogate for insulin resistance, predicted accelerated renal decline in community-dwelling older adults and progression toward end-stage renal disease in a hospital CKD cohort. Part of the effect was conveyed through widened pulse pressure and further amplified by coexisting hypertension, tracing a “metabolism–vascular–renal” cascade (Zhu et al.). Extending these mechanistic insights into clinical practice, a risk score integrating glycemia, blood pressure, lipids, adiposity, and microvascular markers identified patients already losing kidney function at their very first renal reassessment — just six months after baseline. This loss of function preceded the appearance of overt albuminuria (Jiang et al.). The same principle of early detection was taken a step further by a machine-learning study in elderly adults with type 2 diabetes. A model combining clinical indicators, traditional Chinese medicine symptoms, and ultrasound imaging outperformed models based on clinical data alone. The model’s leading predictors were diabetic retinopathy, uric acid, and carotid plaque, which together tie early renal injury to microvascular damage, oxidative stress, and atherosclerosis, echoing the metabolic–vascular–renal axis described above (Fang et al.). Taken together, these studies establish that metabolic dysregulation is an early, detectable, and potentially modifiable driver of renal deterioration.

## Midstream: the vascular–renal bridge

Between the metabolic trigger and end-organ failure lies a vascular–renal bridge through which systemic stress is transmitted to the kidney and the heart. A J-shaped relationship between creatinine and post-PCI adverse events illustrates this cardiorenal interplay. Creatinine fluctuations activate the RAAS, induce oxidative and inflammatory injury to the coronary endothelium, and heighten vulnerability to contrast-induced acute kidney injury. These mechanisms form a chain that converts subtle renal reserve loss into major adverse events (Zhu et al.). At the population level, a network analysis from Guangxi positioned hypertension as the central node connecting cardiovascular, renal, and metabolic conditions, highlighting the bidirectional cross-talk that makes elevated blood pressure both a cause and a consequence of dysfunction across all three systems (Li et al.). At the molecular level, preclinical evidence shows that oxidative stress and chronic inflammation in CKD uncouple the NO–sGC–cGMP signaling pathway. sGC stimulators and activators, by restoring this pathway, lowered blood pressure, reduced kidney weight, and improved creatinine, urea, and uric acid across diverse animal models (Zhang et al.). These observations define the vascular–renal conduit through which upstream metabolic insults reach their target organs.

## The amplifier: immune–inflammatory activation

Inflammation does not merely accompany CKM syndrome — it amplifies it. Metabolic stress and hemodynamic overload sustain a chronic, low-grade inflammatory state that injures the vasculature, the myocardium, and the kidney. Once established, this inflammatory milieu can become self-perpetuating, persisting even after the initiating trigger is controlled. And in its most extreme form it erupts as a lethal cytokine storm. In primary aldosteronism, excess aldosterone activates multiple inflammatory pathways: driving proinflammatory macrophage polarization, disturbing T-cell homeostasis, and engaging the NLRP3 inflammasome and IL-6 signaling. The myocardial fibrosis and vascular remodeling that follow outlast mineralocorticoid receptor blockade, offering a mechanistic account of the residual cardiovascular risk that persists in treated patients (Liu et al.). Moving from hormonal driver to organ-specific consequence, an elevated C-reactive protein-to-lymphocyte ratio selectively predicted proteinuric CKD rather than isolated eGFR decline in a hypertensive cohort, implicating inflammation in glomerular barrier disruption rather than hemodynamic sclerosis. The effect was further amplified when inflammatory burden coexisted with uncontrolled blood pressure (Yang et al.). At the acute extreme, the systemic inflammatory response index tracked 30-day mortality in fulminant myocarditis complicated by acute kidney injury, demonstrating how unchecked immune activation can become rapidly lethal (Zhu et al.).

## Downstream: interventions and outcomes

The final segment of the CKM chain concerns how accumulated damage can be modified. A population-based survey from Guangxi captures the scale of the challenge: over three-quarters of adults fall within CKM stages 1–4, and modifiable factors such as education, diet, alcohol, and hyperuricemia identify realistic prevention targets (Li et al.). Among the most accessible interventions is physical activity. Accelerometry data from over 88,000 UK Biobank participants showed that both light and moderate-to-vigorous physical activity lowered incident CVD risk in a stage-dependent manner. The protective benefit was most pronounced in stages 1 and 2; in stage 3. However, the dose–response curve turned U-shaped, signaling that excessive high-intensity exercise may become counterproductive as disease advances (An et al.). The mechanisms underlying these benefits are multi-layered: a narrative review documented how exercise activates antioxidant defenses, enhances mitochondrial quality control, alleviates endoplasmic reticulum stress, and suppresses inflammatory mediators — supporting stage-adapted programs that span cardiology, nephrology, and rehabilitation (Sun et al.). The case for individualization is reinforced by an apparently paradoxical finding in older adults: lower LDL-C was associated with greater frailty, a “cholesterol paradox” best explained by inflammation-driven lipid remodeling and nutritional vulnerability, cautioning against uniform lipid targets in frail elders (Liu et al.). Together, these contributions argue that downstream management must calibrate the intensity and nature of the intervention to the patient’s disease stage, inflammatory burden, and physiological reserve.

## Conclusions and future directions

Read together, these twelve contributions support a view of CKM cardiovascular risk as a linear yet self-reinforcing pathway. Composite metabolic–inflammatory markers (TyG index, IRD-PS, CLR) appear to outperform isolated measurements for risk prediction (Zhang et al., Zhu et al., Yang et al.). The vascular–renal axis — RAAS activity, arterial stiffness, and NO–sGC–cGMP signaling — deserves recognition as a modifiable intermediate target (Zhu et al., Li et al., Zhang et al.). Inflammation should be treated as an adjustable amplifier across the whole spectrum (Liu et al., Yang et al., Zhu et al.). And both exercise and pharmacotherapy exhibit stage-dependent, pleiotropic effects that favor individualized, phenotype-driven prescribing (An et al., Sun et al.). The principal limitation is methodological: most evidence remains cross-sectional, retrospective, or *post hoc*, so prospective multicenter studies with standardized staging and longitudinal follow-up are required, together with deeper mechanistic work on aldosterone’s non-genomic signaling and the pathways from inflammation to podocyte and endothelial damage. We hope this second volume encourages continued investigation and, ultimately, evidence-based care that interrupts the chain before it reaches its end.
